# Long non-coding RNA ROR recruits histone transmethylase MLL1 to up-regulate TIMP3 expression and promote breast cancer progression

**DOI:** 10.1186/s12967-020-02682-5

**Published:** 2021-03-02

**Authors:** Aixia Hu, Fan Hong, Daohong Li, Yuwei Jin, Lingfei Kon, Ziguang Xu, Hui He, Qi Xie

**Affiliations:** 1 Department of Pathology, Henan Provincial People’s Hospital, No. 7, Weiwu Road, Zhengzhou, 450003 Henan People’s Republic of China; 2 Henan University People’s Hospital, Zhengzhou, 450003 People’s Republic of China

**Keywords:** Breast cancer, Long non-coding RNA ROR, Mixed-lineage leukemia 1, Tissue inhibitors of metalloproteinase 3, Methylation, Proliferation, Invasion

## Abstract

**Background:**

As a significant cause of cancer deaths worldwide, breast cancer continues to be a troublesome malignancy. Long non-coding RNAs (lncRNAs) have been implicated in the development of breast cancer. Abnormal methylation has been associated with unfavorable breast cancer prognosis. Herein, the current study aimed to elucidate the role of lncRNA ROR in breast cancer.

**Methods:**

RT-qPCR was performed to determine whether lncRNA ROR was highly expressed in breast cancer tissues, while lncRNA ROR expression was detected in both the nuclear and cytoplasm of breast cancer cells. MCF-7 cells were subsequently introduced with oe-lncRNA ROR, sh-lncRNA ROR to explore the effects of lncRNA ROR on cell proliferation, invasion and apoptosis.

**Results:**

RIP, RNA pull-down and ChIP assays provided evidence suggesting that lncRNA ROR recruited transmethylase MLL1 to promote H3K4 trimethylation that enhanced TIMP3 transcription. The rescue experiments demonstrated that lncRNA ROR knockdown could inhibit the progression of breast cancer via the downregulation of TIMP3. Finally, the in vivo experiment findings consistently highlighted the suppressive effects of lncRNA ROR silencing on tumor growth.

**Conclusion:**

Taken together, our study demonstrates that silencing of lncRNA ROR inhibits breast cancer progression via repression of transmethylase MLL1 and TIMP3, emphasizing the potential of lncRNA ROR as a novel target against breast cancer.

## Background

Breast cancer remains a significant malignant tumor mainly afflicting the female population, with studies highlighting its rank in the top three most common cancers worldwide along with colon and lung cancer [1]. Breast cancer has been emphasized as a chief cause of cancer diagnoses among women worldwide, representing approximately 15% of all cancer deaths and 25% of all diagnosed cancers in 2012 [2]. Patients that survive breast cancer are often left to endure the chronic iatrogenic effects of therapy, such as infertility, vasomotor symptoms, lymphedema, and constant pain and fatigue [3]. Therapeutic strategies for breast cancer are often dependent on the characteristics of the tumor, including the tumor location, size and the tumor stage [4]. Significant improvements in relation to breast cancer diagnostic screening methods have led to improved breast cancer survival rates in recent years, while breast cancer recurrence rates are often found in the same or contralateral breast [5]. Serum DNA methylation has been reported to be a powerful predictor of survival outcomes and a promising clinical marker of metastatic breast cancer [6]. Accumulating studies continue to emphasize long non-coding RNAs (lncRNAs) as tumor inhibitors or carcinogenic factors in breast cancer, highlighting their potential as therapeutic targets for breast cancer [7].

LncRNAs can be defined as a type of short RNAs with a length over 200 nucleotides, which are both independently transcribed and tissue-specific [8]. LncRNAs have been reported to exert a wide array of effects on various biological processes and their aberrant expression is linked to various diseases, including cancers [9]. LncRNA ROR is a newly recognized lncRNA that has been linked with tumor formation, development and metastasis of various cancers including breast cancer [10]. LncRNA ROR inhibition has been reported to trigger autophagy in breast cancer to overturn resistance to Tamoxifen [11]. Besides, lncRNA ROR has been suggested to promote tumorigenesis through attracting gene-specific histone methylation [12]. Mixed-lineage leukemia 1 (MLL1), a methyltransferase of histone lysine 4 (H3K4), has been speculated to provide support for gene transcription and its rearrangements cause fusion protein expression without H3K4 methylation activity [13]. The MLL family has been implicated in the progression of breast cancer [14]. TIMP3 represents a crucial component in the family of tissue inhibitors of metalloproteinase (TIMPs), which is primarily secluded to tissue extracellular matrix and suppresses sheddases, transmembrane MMPs and membrane bound MMPs [15]. TIMP3 promoter methylation has been newly identified as an epigenetic candidate for BRCA1ness breast cancer therapy [16]. Following the aforementioned exploration of literature, we put forth the hypothesis that lncRNA ROR acts as an oncogene in breast cancer via its regulation of MLL1 and TIMP3. We aimed to elucidate the regulatory mechanism by which lncRNA ROR contributes to the progression of breast cancer in connection with MLL1 and TIMP3, with the intention of presenting a theoretical foundation for an enhanced understanding of breast cancer treatment.

## Materials and methods

### Ethics statement

Written informed consent documentation was obtained from all participants prior to enrollment into the study. The study protocols were performed with the approval of the Ethics Committee of Henan Provincial People’s Hospital and conducted in strict accordance with the Declaration of Helsinki of ethical principles for medical research involving human subjects. All animal experiments were performed in strict adherence with the recommendations in the Guide for the Care and Use of Laboratory Animals of the National Institutes of Health. The protocol of animal experiments was approved by the Institutional Animal Care and Use Committee of Henan Provincial People’s Hospital. Animal experiments were performed on the basis of ensuring minimal animal number of use and minimizing the degree of pain inflicted during experiments.

### Study subjects

Breast cancer tissues and adjacent tissues were collected from 60 patients diagnosed with primary breast cancer undergoing surgical treatment at the Henan Provincial People’s Hospital from June 2016 to December 2107. The tissues were frozen in liquid nitrogen and subsequently stored in a -80℃ refrigerator for reverse transcription quantitative polymerase chain reaction (RT-qPCR) and immunohistochemistry. All enrolled patients were females aged 27 to 69 years with a mean age of 49 years. All patients had their diagnosis pathologically confirmed with none of the patients enrolled with a history of neoadjuvant chemotherapy prior to surgery. As per the international tumor node metastasis (TNM) clinical stage of breast cancer, there were 11 cases at stage I (18.33%, 11/60), 40 cases at stage II (66.67%, 40/60) and 9 cases at stage III (15.00%, 9/60).

### Cell culture

Breast cancer cells MCF-7, MDA-MB-231 (Shanghai Zhongqiaoxinzhou Biotechnology Co., Ltd., Shanghai, China), SKBR3 [American Type Culture Collection (ATCC), Manassas, VA, USA], BCAP (Procell Life Science & Technology Co., Ltd., Wuhan, Hubei, China) and normal mammary epithelial cells MCF-10A (ATCC, Manassas, VA, USA) were cultured with Dulbecco’s modified eagle medium (DMEM) supplemented with 10% fetal bovine serum (FBS) at 37 ℃ with 5% CO_2_. After adherence had been confirmed, the cells were passaged and treated with 0.25% trypsin (Hyclone company, Logan, UT, USA). Cells exhibiting logarithmic growth were collected for later use. RT-qPCR was performed in order to select the cell line with highest lncRNA ROR expression among MCF-10A, MCF-7, SKBR3, BCAP and MDA-MB-231 for subsequent experimentation.

### Fluorescent in situ hybridization (FISH) assay

FISH kit (C10910, Guangzhou RiboBio Co., Ltd., Guangdong, China) was employed to detect lncRNA ROR expression in MCF-7 cells. The cell slides were placed at the bottom of a 24-well plate where MCF-7 cells at the logarithmic growth phase were detached (about 6 × 10^4^ cells/well). Upon reaching 60–70% cell confluence, the cells were washed once with phosphate buffer saline (PBS) for 5 min and fixed with 4% paraformaldehyde at room temperature for 10 min. One mL of precooling penetrating fluid was then added to each well and permitted to stand at 4 ℃ for 5 min. Two-hundred μL of prehybridization solution was added to each well and sealed at 37 ℃ for 30 min. Hybridization solution was subsequently preheated at 37 ℃ and added with 2.5 μL of 20 μM FISH Probe Mix stock solution under conditions void of light. After the prehybridization solution had been discarded, probe hybridization solution was added for hybridization at 37 ℃ overnight under dark conditions. The cells were subsequently rinsed with lotion I 3 times (5 min per time) at 42 ℃ under conditions void of light in order to limit background signal followed by rinsing with lotion II and lotion III at 42 ℃ under dark conditions. The cells were subsequently stained with 4′,6-diamidino-2-phenylindole (DAPI) dye liquor for 10 min under dark conditions followed by 3 rinses with phosphate buffered saline (PBS) (5 min per wash). Finally, the cell slides were carefully removed from each well and fixed in the glass slides by mounting medium for fluorescence detection. The specific lncRNA ROR probes were synthesized by Guangzhou RiboBio Co., Ltd. (Guangdong, China).

### RNA pull-down assay

The lncRNA ROR was transcribed in vitro using T7 RNA polymerase (Ambion, Austin, TX, USA) and subsequently purified using a RNeasy Plus Mini Kit (QIAGEN, Dusseldorf, Germany) and DNase I (QIAGEN, Dusseldorf, Germany). The purified RNA 3′ was labeled with a biotin RNA labeling mixture (Ambion, Austin, TX, USA). Next, 1 μg of labeled RNA was added in RNA structure buffer (10 mmol/L Tris pH7, 0.1 mmol/L KCl, 10 mmol/L MgCl_2_) and heated to 95 °C, after 2 min. The mixed buffer was incubated on ice for 3 min, and then allowed to stand at room temperature for 30 min. Next, 3 μg of breast cancer cells were lysed using cell lysis buffer (Sigma-Aldrich, St. Louis, MO, USA) at 4 °C for 1 h. The cell lysate was centrifuged at 12,000×*g* at 4 °C for 10 min, with the supernatant collected and transferred to an RNase-free centrifuge tube. Next, 400 ng of biotinylated RNA was added to 500 μL of RNA binding protein immunoprecipitation (RIP) buffer and mixed with cell lysate for 1 h at room temperature. Streptavidin magnetic beads were added to each binding reaction and collectively incubated at room temperature for 1 h. Finally, the cells were washed 5 times with RIP buffer, and incubated with 5 × loading buffer at 95 °C for 5 min. The eluted MLL1 protein was detected by Western blot assay.

### RNA binding protein immunoprecipitation (RIP) assay

The binding of lncRNA ROR to MLL1 protein was detected using a RIP kit (Merck Millipore, Billerica, MA, USA) [17]. The cells were washed with pre-cooled PBS after which the supernatant was discarded. The cells were lysed with radioimmunoprecipitation assay lysis buffer (P0013B, Beyotime, Shanghai, China) in an ice bath for 5 min, and centrifuged at 12,000×*g* for 10 min at 4 °C. The cell lysate was incubated and co-precipitated with the antibody. Specifically, 50 μL of magnetic beads were resuspended in 100 μL of RIP wash buffer, and mixed with 5 μg of antibody. The magnetic bead-antibody complex was resuspended in 900 μL of RIP wash buffer and incubated with 100 μL of cell lysate at 4 °C overnight. The RNA was extracted from magnetic bead-protein complex for subsequent PCR detection after detachment with proteinase K. The antibody used for RIP included rabbit anti-MLL1 (1: 200, # 61,702, Cell Signaling Technology, Danvers, MA, USA), and immunoglobulin G (IgG, ab109489, 1: 100, Abcam, Cambridge, MA, USA) in the negative control.

### Chromatin immunoprecipitation (ChIP)

The histone-3 lysine-4 trimethylation (H3K4me3) in TIMP3 promoter region was detected using a ChIP assay [18]. The breast cancer cells were fixed with 1% formaldehyde after cell confluence achieved 70—80%, with the DNA and protein of the cells cross-linked for 10 min at room temperature. After cross-linking, the cells were ultrasonicated for 10 s, followed by 15 repeats at 10 s intervals. The collected fragments were then centrifuged at 13,000 rpm at 4 °C, after which the supernatant was collected into two tubes and incubated with specific antibody mouse polyclonal anti-H3K4me3 (1:100, ab8580, Abcam, Cambridge, UK) or antibody rabbit monoclonal anti-IgG (1:100, ab109489, Abcam, Cambridge, UK) in the negative control overnight at 4 °C, with the endogenous DNA–protein complex precipitated by Protein Agarose/Sepharose. After centrifugation, the supernatant was discarded. The non-specific complex was cross-linked at 65 °C overnight, with the DNA fragment was purified by phenol/chloroform extraction. The content of the TIMP3 promoter fragment was detected by qPCR.

### Preparation and infection of lentivirus

The overexpression (oe)-lncRNA ROR, short hairpin TIMP3 (sh-TIMP3) and sh-lncRNA ROR were synthesized by Shanghai Sangon Biological Engineering Technology & Services Co., Ltd. (Shanghai, China), which were subsequently co-transfected with three package vectors PMDLG/PRRE, vesicular stomatitis virus glycoprotein (VSVG), and respiratory syncytial virus (RSV)/respiratory entericorphan virus (REV) into HEK293T cells. After 6 h, the culture medium was replaced with fresh DMEM containing 10% FBS. The lentivirus-containing medium was harvested at both the 48 and 72 h time points post-transfection and filtered to remove cell debris using a 10 mL syringe and a 0.45 μm low protein binding filter (Millipore, Billerica, MA, USA). The virus was either immediately used for infection or stored at − 80℃ for later use. Regarding infection, the medium containing lentivirus was employed to infect the MCF7 cells in the presence of 5 μg/mL polybrene. The plates were subsequently placed in a 37℃ incubator with 5% CO_2_ for 20–24 h. The old medium was renewed with fresh complete medium. Finally, the infected MCF7 cells were analyzed using western blot analysis or RT-qPCR.

### RT-qPCR

Total RNA was extracted in accordance with the instructions of the miRNeasy Mini Kit (Qiagen company, Hilden, Germany). Next, 5 μL RNA specimens were diluted by 20 times with RNA-free enzyme ultrapure water. Absorption values at 260 nm and 280 nm was read using an ultraviolet spectrophotometer. The RNA concentration and purity were then determined. An OD260/OD280 ratio between 1.7 and 2.1 was considered to be indicative of high purity for subsequent experiments. Reverse transcription reaction was conducted using a PCR amplifier (9700, Beijing Dingguo Changsheng Biotechnology co. Ltd, Beijing, China) to synthesize cDNA template as per the instructions specified in the EasyScriPt First-Strand cDNA Synthesis SuPerMix manual (AE301-02, Beijing TransGen Biotech Co., Ltd., Beijing, China). The GAPDH, lncRNA ROR and TIMP3 primers (Table 1) were designed and synthesized by Shanghai Sangon Biological Engineering Technology & Services Co., Ltd. (Shanghai, China). cDNAs were collected for real-time fluorescent quantitation PCR on a 7500 fluorescent quantitation PCR instrument (ABI Compfany, Oyster Bay, NY, USA) based on the specifications of SYBR®Premix Ex Taq™ II kit (Takara, Dalian, Liaoning, China). Glyceraldehyde-3-phosphate dehydrogenase (GAPDH) was regarded as the internal reference, with the 2^−ΔΔCt^ method applied to express the ratio of the target gene expression in the experimental group to that of the control group [19].

**Table 1:** Primer sequences for RT-qPCR

Gene	Primer sequence
GAPDH	F: 5′-CACCCATCACAAACATGGGTGCAT-3'
	R: 5′- TTTCAGGAAATGAAGCCTGCCAGC-3'
LncRNA ROR	F: 5′-AACAGTAGAGTGGGGCCTGA-3'
	R: 5′-GTGGCACACTGCTCAAAACC-3'
TIMP3	F: 5′-TCTCTGTGGCCTYAAGCTGGA-3'
	R: 5′-CCGTGTACATCTTGCCATCATAGA-3'

RT-qPCR, reverse transcription quantitative polymerase chain reaction; F, forward; R, reverse; GAPDH, glyceraldehyde-3-phosphate dehydrogenase; LncRNA ROR, long non-coding RNA ROR; TIMP3, tissue inhibitors of metalloproteinase 3

### Western blot analysis

The MCF-7 cells were added with prepared radio-immunoprecipitation assay (RIPA) lysis buffer (Beyotime Biotechnology Co., Ltd., Shanghai, China). The cells were then transferred into a 1.5 mL EP tube and fully lysed. The supernatant was collected after centrifugation at 14,000×*g* for 10 min. The bicinchoninic acid (BCA) method was applied to determine protein concentration. A sodium dodecyl sulfate-polyacrylamide gel electrophoresis (SDS-PAGE) kit was employed to prepare 10% separation gel and 5% spacer gel. The protein was subsequently separated by SDS-PAGE, followed by wet transfer onto a nitrocellulose membrane which was sealed with 5% bovine serum albumin (BSA) at room temperature for 1 h. The membrane was then probed with diluted primary rabbit polyclonal antibodies to MLL1 (1:800, # 61702, Cell Signaling Technology, Danvers, MA, USA), TIMP3 (ab39184, 1:1000), Vimentin (ab45939, 1 μg/mL), E-cadherin (ab15148, 1:500), N-cadherin (ab18203, 1 μg/mL), matrix metalloproteinase (MMP)-2 (ab37150, 1 μg/mL), MMP-9 (ab73734, 1 μg/mL), proliferating cell nuclear antigen (PCNA) (ab18197, 1 μg/mL), Bcl-2 associated X (Bax) (ab53154, 1:1000), B-cell lymphoma 2 (Bcl-2) (ab196495, 1:1000), Cleaved caspase-3 (ab2302, 1 μg/mL) and GAPDH (ab37168, 1 μg/mL) and rabbit monoclonal antibody Ki67 (ab16667, 1 μg/mL) at 4 °C overnight. All the aforementioned antibodies were purchased from Abcam (Cambridge, MA, USA). On the second day, the membrane was washed with phosphate buffered saline with tween-20 (PBST) 3 times (10 min per wash) and incubated with 5% skim milk-diluted secondary goat anti-rabbit polyclonal antibody (ab7312, Abcam, Cambridge, MA, USA) at room temperature for 1 h. After three additional PBST washes (10 min per wash), the membrane was added with electrogenerated chemiluminescence (ECL) solution (BLH02S050, Bioworld, Atlanta, GA, USA) and visualized by the bio-Rad gel imaging system (MG8600, Beijing Thmorgan Biotechnology Co., Ltd., Beijing, China). IPP7.0 software (Media Cybernetics, Silver Spring, MD, USA) was utilized for quantitative analysis.

### 5-Ethynyl-2′-deoxyuridine (EdU) staining

Next, 120 μL matrigel was added to each well in the 24-well plate and incubated at 37℃ for 30 min. The cells were then detached with 0.25% trypsin and dispersed into single cell suspension at density of 1.6 × 10^4^ cells/mL with DMEM/F12 Mixture medium containing 2% serum. Each well was added with 250 μL cell suspension and incubated at 37℃ for 48 h. EdU dye liquor was added 18 h prior to cell harvest. Matrigel was triturated by a 200 μL pipette head and uniformly smeared on the cover glasses. After adequate drying, the cover glasses were fixed with 4% paraformaldehyde at room temperature for 30 min, incubated with 0.5% Triton X-100 at room temperature for 25 min, treated with Cu^+^ and trinitride compound solution and DAPI (Beyotime Biotechnology Co., Ltd., Shanghai, China) at room temperature in darkness for 30 min and 5 min respectively, followed by the dropwise addition of anti-quenching solution and mounted. Finally, laser scanning confocal microscope (FV300, Olympus Optical Co., Ltd., Tokyo, Japan) was used to obtain images. Cells exhibiting a nucleus stained with EdU and DAPI were considered to be positive proliferation cells. The average value of each 3 wells was calculated, with each experiment repeated 3 times.

### Transwell assay

Matrigel (Becton, Dickinson and Company, NJ, USA) was diluted by precooling serum-free DMEM at a ratio of 1:10. The apical chamber was subsequently added with 100 μL diluted matrigel and permitted to stand at room temperature for 2 h. The cells were then trypsinized, re-suspended by serum-free DMEM, counted and diluted into density of 3 × 10^5^ cells/mL. Afterwards, 100 μL suspension was added to the apical chamber of the Transwell chamber (Corning Glass Works, Corning, N.Y., USA), and 600 μL of 10% serum-free DMEM (serum as the chemotactic factor) added to the basolateral chamber according to the instructions of Transwell chamber. The cells were then stained crystal violet, and three fields were randomly selected under an optical microscope to count the number of cells across the membrane.

### Flow cytometry

Annexin V-fluorescein isothiocyanate/propidium iodide (FITC/PI) staining kit (556547, Shanghai Shuo Jia Biotechnology Co., Ltd., Shanghai, China) was employed to detect MCF-7 cell apoptosis. Deionized water was used to dilute 10× binding buffer into 1× binding buffer. After centrifugation at 715×*g* at room temperature for 5 min, the MCF-7 cells were re-suspended by precooling 1× PBS and centrifuged at 715×*g* for 5–10 min. The cells were then re-suspended by 300 µL 1× binding buffer and incubated with 5 µL Annexin V-FITC at room temperature for 15 min under dark conditions. The cells were then added with 5 µL PI 5 min prior to the use of a flow cytometer (Cube6, Partec, Leipzig, Germany) and ice-bathed under conditions void of light for 5 min. FITC was detected at the excitation wavelength of 480 nm and 530 nm and PI was measured at over 575 nm.

### Tumor xenograft in nude mice

A total of 24 BALB/C nude mice (aged at 4 weeks;  weighing 18–22 g; sex unlimited, Hunan SLAC Laboratory Animal Co., Ltd., Changsha, Hunan, China) were fed under a controlled specific pathogen-free (SPF) environment. The nude mice were assigned into two groups (12 mice per group). The MCF-7 cells were treated with lentivirus-transduced sh-lncRNA ROR and sh-NC. After the mice had been administered with narcotics via intraperitoneal injection with 2% pentobarbital sodium (0.6 mL/100 g), 0.2 mL cell suspension (10^7^ cells/mL) was subcutaneously injected into the mice. The tumor volume was monitored on a weekly basis. The tumor volume was calculated in accordance with the following formula: V = length × width^2^/2. The nude mice were euthanized at the end of third week, followed by tumor collection.

### Statistical analysis

All data were analyzed using a Statistic Package for the Social Science (SPSS) 21.0 statistical software (IBM Corp. Armonk, NY, USA). Measurement data were expressed as the mean ± standard deviation. Normal distribution and homogeneity of variance tests were initially conducted. With normal distribution and homogeneity of variance, comparisons between groups were conducted by paired* t* test, while those with skewed distribution heterogeneity of variance were by independent sample* t* test. Comparisons among two groups analyzed by independent sample *t* test, and comparisons among multiple groups were analyzed by one-way analysis of variance (ANOVA) or repeated measures ANOVA. Post hoc test was performed using Tukey's test. The level of significant difference was regarded as *p* < 0.05.

## Results

### LncRNA ROR overexpresses in breast cancer tissues

**Fig. 1:**
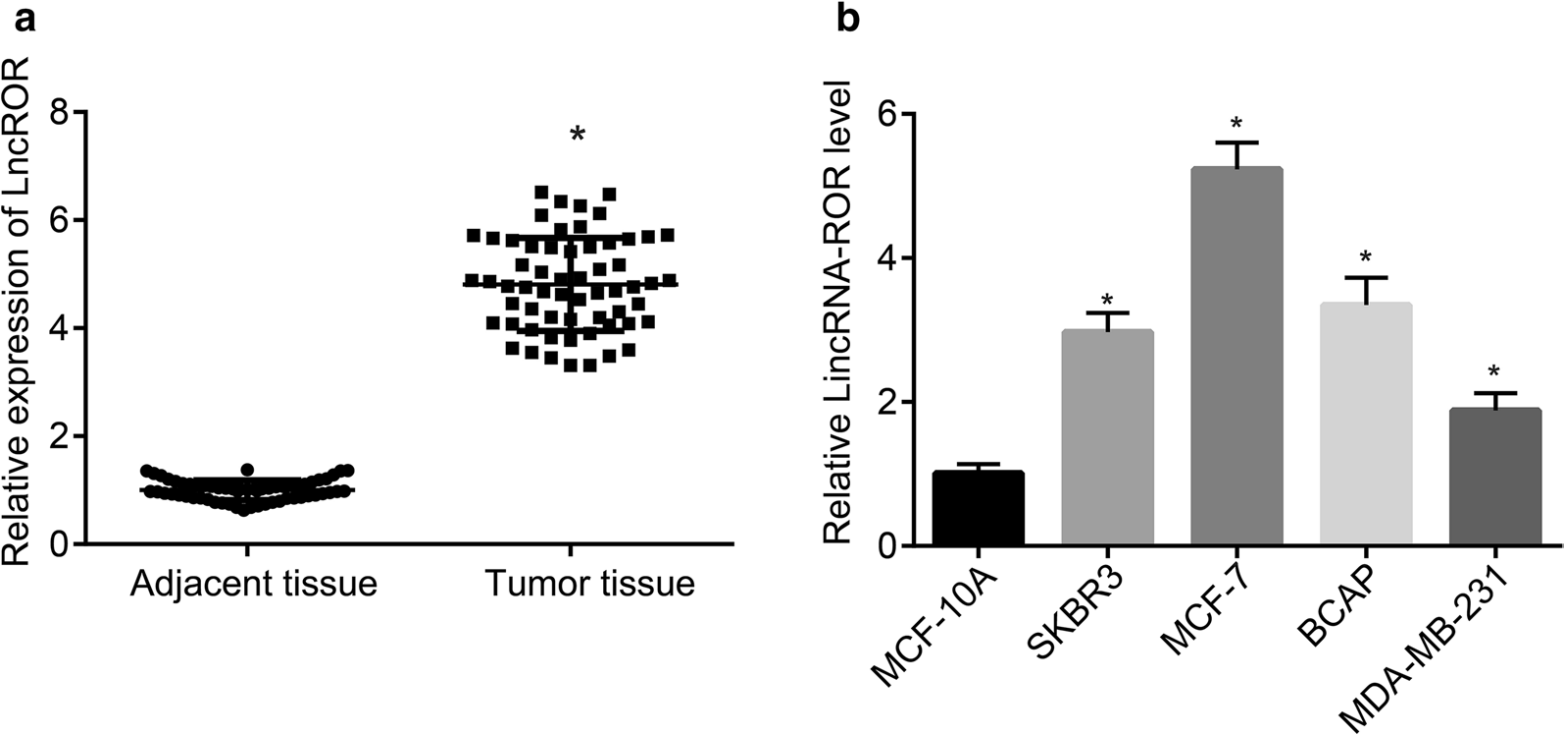
LncRNA ROR is highly expressed in breast cancer tissues. **a** The expression of lncRNA ROR in breast cancer tissues and adjacent tissues detected by RT-qPCR. **b** The expression of lncRNA ROR in normal mammary epithelial cell line MCF-10A and breast cancer cell lines MCF-7, SKBR3, BCAP and MDA-MB-231 determined by RT-qPCR. * *p* < 0.05 vs. the adjacent tissues. The above data are measurement data and expressed as mean ± standard deviation. Comparisons between two groups are analyzed by paired *t* test, and comparisons among multiple groups are analyzed by analysis of variance. Post hoc test was conducted using Tukey’s test. n = 60. The experiment is repeated 3 times. RT-qPCR, reverse transcription quantitative polymerase chain reaction; TIMP3, tissue inhibitors of metalloproteinase 3; n, number

LncRNA ROR has been reported to be involved in the process of tumorigenesis [20]. Hence, to examine the effect of lncRNA ROR in breast cancer, RT-qPCR and immunohistochemistry were performed to characterize its expression in breast cancer tissues and adjacent tissues. The results obtained indicated that the expression of lncRNA ROR and TIMP3 was higher in breast cancer tissues than that in the adjacent tissues (*p* < 0.05; Fig. 1a). RT-qPCR was subsequently performed to select the cell line with highest expression of lncRNA ROR among MCF-10A, MCF-7, SKBR3, BCAP and MDA-MB-231. MCF-10A is a human normal mammary epithelial cell line, and MCF-7 is a human breast cancer cell line with characteristics of metastasis. MDA-MB-231 exhibited a stronger metastatic capacity than that of SKBR3 and BCAP. The RT-qPCR results suggested that MCF-7 expressed the highest level of lncRNA ROR relative to the aforementioned cell lines (Fig. 1b). Thus, MCF-7 was used for the following experiments.

### Silencing of lncRNA ROR inhibits cell proliferation and invasion, promotes apoptosis in breast cancer

**Fig. 2:**
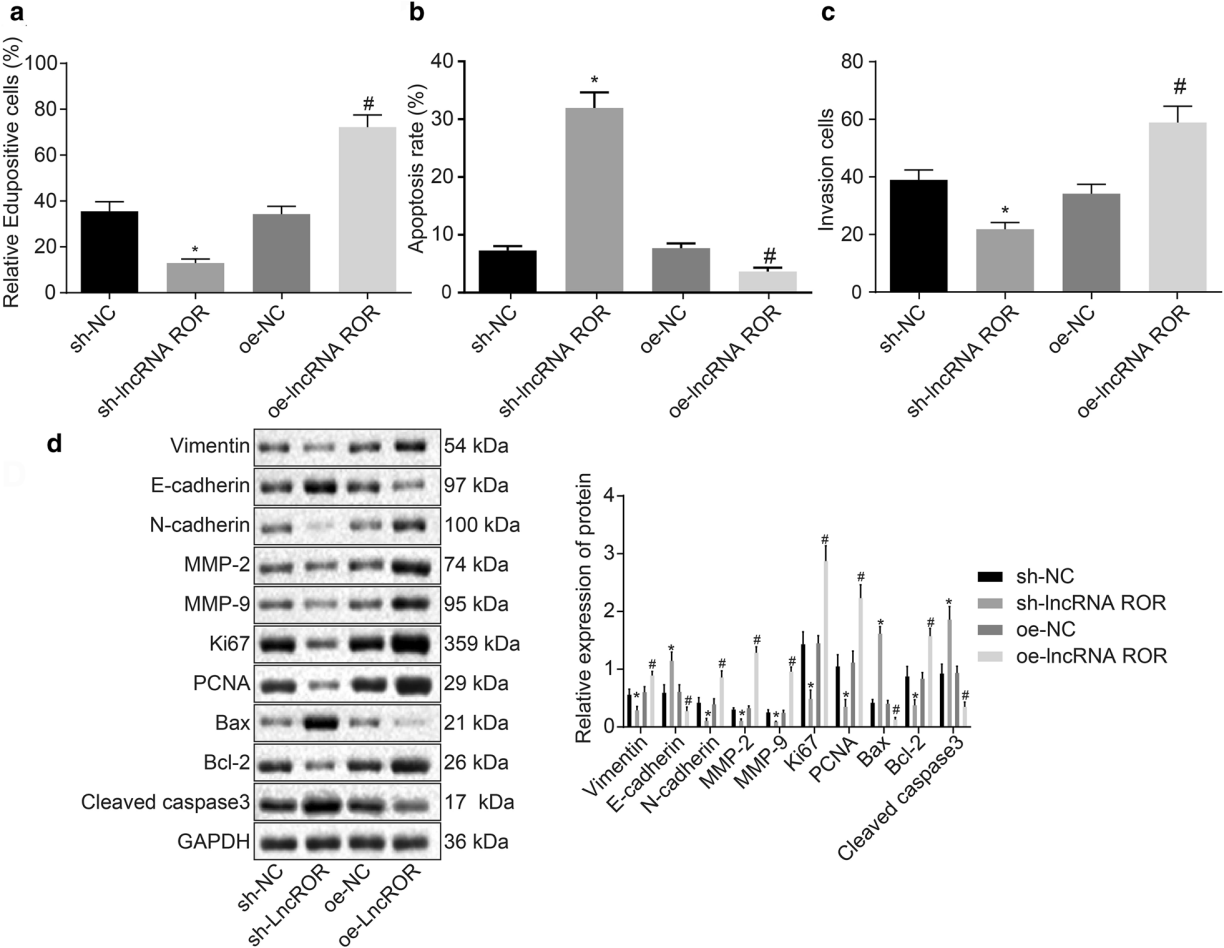
LncRNA ROR silencing inhibits proliferation and invasion, and promotes apoptosis of breast cancer cells. MCF-7 cells were treated with sh-lncRNA ROR or oe-lncRNA ROR. **a** Cell proliferation of MCF-7 detected by EdU. **b** Cell apoptosis and cycle of MCF-7 measured by flow cytometry. **c** Cell invasion of MCF-7 assessed by Transwell assay. **d** Protein levels of Vimentin, N-cadherin, MMP-2, MMP-9, Ki67, PCNA, Bcl-2, E-cadherin, Bax and Cleaved caspase-3 in MCF-7 determined by Western blot analysis. *p < 0.05 vs*.* MCF-7 cells treated with sh-NC. ^#^*p* < 0.05 vs. MCF-7 cells treated with oe-NC. The above data are measurement data and expressed as mean ± standard deviation. Comparisons among multiple groups are analyzed by independent sample *t* test. The experiment is repeated 3 times. EdU, 5-Ethynyl-2′-deoxyuridine; MMP, matrix metalloproteinase; PCNA, proliferating cell nuclear antigen; Bax, Bcl-2-associated protein x; Bcl-2, B cell lymphoma 2; NC negative control

Next, to further ascertain the effect of lncRNA ROR on cell biological functions, a series of in vitro experiments including EdU, Transwell assay and flow cytometry as well as Western blot assay were conducted. The data obtained demonstrated that treatment with sh-lncRNA ROR decreased cell proliferation (Fig. 2a) and invasion (Fig. 2b), while resulting in elevated apoptosis (Fig. 2c) as well as reduced protein levels of Vimentin, N-cadherin, MMP-2, MMP-9, Ki67, PCNA and Bcl-2 and increased protein levels of E-cadherin, Bax and Cleaved caspase-3 (Fig. 2d). Treatment with oe-lncRNA ROR was found to reverse the aforementioned changes (Fig. 2a–d). The results indicate that silencing of lncRNA ROR inhibits cell proliferation and invasion, while promoting breast cancer cell apoptosis.

### LncRNA ROR promotes H3K4me3-induced TIMP3 transcription by recruiting MLL1

**Fig. 3:**
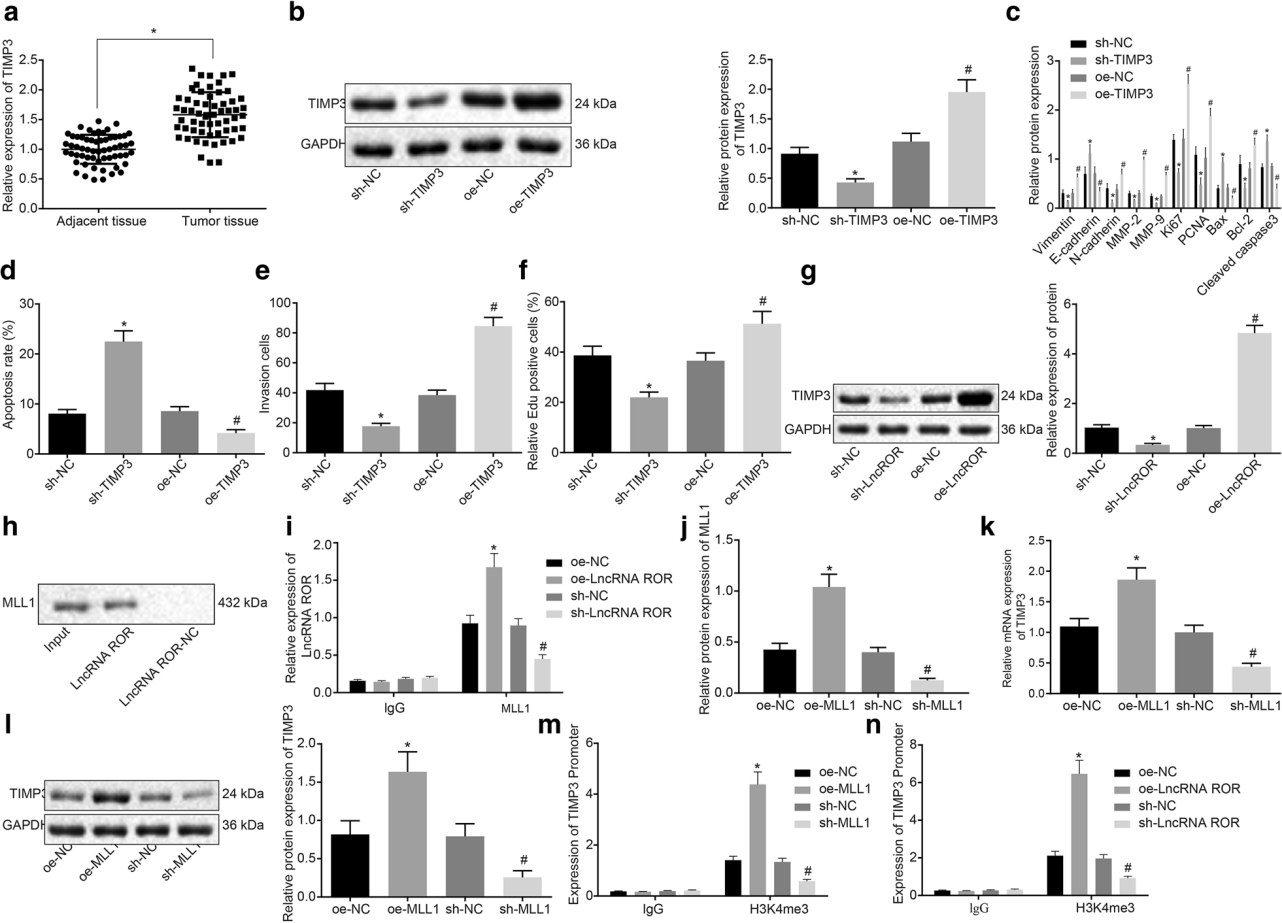
LncRNA ROR promotes H3K4m3 on TIMP3 by recruiting MLL1, thus further promoting the transcription of TIMP3. MCF-7 cells were treated with sh-TIMP3/oe-TIMP3 and sh-lncRNA ROR/oe-lncRNA ROR. **a** The mRNA expression of TIMP3 in breast cancer tissues and adjacent normal tissues determined by RT-qPCR. *p < 0.05 vs. the adjacent normal tissues. **b** The protein expression of TIMP3 after MCF-7 cells were transfected with sh-TIMP3 or oe-TIMP3 determined by Western blot analysis. **c** The protein levels of Vimentin, N-cadherin, MMP-2, MMP-9, Ki67, PCNA, Bcl-2, E-cadherin, Bax and Cleaved caspase-3 in MCF-7 cells transfected with sh-TIMP3 or oe-TIMP3 determined by Western blot analysis. **d** Apoptosis of MCF-7 cells transfected with sh-TIMP3 or oe-TIMP3 measured by flow cytometry. **e** Invasion of MCF-7 cells transfected with sh-TIMP3 or oe-TIMP3 measured by Transwell assay. **f** Proliferation of MCF-7 cells transfected with sh-TIMP3 or oe-TIMP3 measured by EdU assay. **b**–**f** *p < 0.05 vs. that of the cells transfected with sh-NC, ^#^*p* < 0.05 vs. that of the cells transfected with oe-NC. **g** The protein expression of TIMP3 after MCF-7 cells were transfected with sh-lncRNA ROR or oe-lncRNA ROR determined by Western blot analysis. **h** The localization of lncRNA ROR in MCF-7 cells detected by FISH assay. **i** The MLL1 enrichment after lncRNA ROR overexpression and silencing detected by RNA-pull-down assay. **j** The level of lncRNA ROR pulled-down with MLL1 determined by RIP assay. **k** The expression of TIMP3 in MCF-7 cells transfected with oe-MLL1 or sh-MLL1 measured by RT-qPCR. **l** The expression of TIMP3 in MCF-7 cells transfected with oe-MLL1 or sh-MLL1 measured by Western blot assay. **m** The expression of TIMP3 in MCF-7 cells transfected with oe-MLL1 or sh-MLL1 measured by qPCR after ChIP assay using antibody to H3K4me3. **n** The expression of TIMP3 in MCF-7 cells transfected with oe-lncRNA ROR or sh-lncRNA ROR measured by qPCR after ChIP assay using antibody to H3K4me3. In **j**–**p** *p < 0.05 vs. that of the cells transfected with sh-NC, ^#^*p* < 0.05 vs. that of the cells transfected with oe-NC. The above data are measurement data and expressed as mean ± standard deviation. Comparisons among multiple groups are analyzed by independent sample *t* test. The experiment is repeated 3 times. RT-qPCR, reverse transcription quantitative polymerase chain reaction; TIMP3, tissue inhibitors of metalloproteinase 3; EdU, 5-Ethynyl-2′-deoxyuridine; MMP, matrix metalloproteinase; ChIP, Chromatin immunoprecipitation; FISH, Fluorescent in Situ Hybridization; PCNA, proliferating cell nuclear antigen; Bax, Bcl-2-associated protein x; Bcl-2, B cell lymphoma 2; NC negative control

According to the prediction at online website (http://pridb.gdcb.iastate.edu/RPISeq/), lncRNA can recruit methyltransferase MLL1, and MLL1 can be used as a specific histone methyltransferase that trimethylates H3K4 [21]. The prediction provided by another online website (http://genome.ucsc.edu/) highlighted the presence of H3K4m3 on the TIMP3 gene, as well as the loss of TIMP3 which is a tumor suppressor in breast cancer [22]. Thus, we hypothesized that lncRNA ROR could promote the H3K4m3 on TIMP3 gene by recruiting methyltransferase MLL1, ultimately promoting the transcription of TIMP3. In order to verify this hypothesis, the expression of TIMP3 in breast cancer tissues and adjacent normal tissues was initially determined by RT-qPCR. In comparison to the adjacent tissues, the expression of TIMP3 in breast cancer tissues was increased (Fig. 3a). TIMP3 was overexpressed and silenced in MCF-7 cells by transfection with sh-TIMP3 and oe-TIMP3, respectively. The results of Western blot assay revealed that the TIMP3 protein expression was markedly decreased after sh-TIMP3 transfection, while elevated following oe-TIMP3 transfection (Fig. 3b). Next, compared with the cells transfected with sh-NC, the proliferation and invasion ability of breast cancer cells transfected with sh-TIMP3 were dramatically decreased, while apoptosis was notably increased (Fig. 3c–f). On the contrary, overexpression of TIMP3 resulted in enhanced proliferation and invasion abilities and inhibited apoptosis (Fig. 3c–f). Therefore, it is suggested that TIMP3 exhibited oncogenic role in breast cancer cells.

Next, to explore the relationship between lncRNA ROR and TIMP3, the expression of TIMP3 was determined following the overexpression and silencing of lncRNA ROR with the results suggesting that sh-lncRNA ROR reduced the expression of TIMP3, and oe-LncRNA ROR elevated the expression of TIMP3 (*p* < 0.05) (Fig. 3g). The FISH experiment results indicated that lncRNA ROR was expressed in the nuclei and cytoplasm of MCF-7 cells (Fig. 3h). RNA pull-down and RIP assays were performed to verify whether lncRNA ROR could recruit MLL1 in breast cancer, which displayed that lncRNA ROR pulled down MLL1 (Fig. 3i), and after overexpression of lncRNA ROR, the expression of lncRNA ROR enriched on MLL1 was notably elevated. Following the silencing lncRNA ROR, the enrichment of lncRNA ROR on MLL1 was markedly reduced (Fig. 3j). MLL1 was subsequently overexpressed and silenced in MCF-7 cells and found that MLL1 and TIMP3 expression significantly increased after overexpression of MLL1 and significantly decreased after knockdown of MLL1 (Fig. 3k, l). The enrichment of H3K4me3 on TIMP3 promoter was detected by ChIP assay. The results demonstrated that enrichment of H3K4me3 on TIMP3 promoter was increased following the overexpression of MLL1, while H3K4me3 enrichment on the TIMP3 promoter was reduced after silencing MLL1 (Fig. 3m). The experimental results suggested that MLL1 promoted H3K4 methylation and further increased TIMP3 expression. Following the overexpression and silencing of lncRNA ROR in breast cancer, ChIP was performed to detect the enrichment of H3K4me3 on the TIMP3 promoter. The H3K4me3 enrichment on the TIMP3 promoter was elevated after overexpression of lncRNA ROR but was reduced after silencing lncRNA ROR (Fig. 3n). Altogether, the aforementioned results suggested that lncRNA ROR can recruit MLL1 to promote H3K4 methylation, thereby increasing the expression of TIMP3.

### TIMP3 silencing reversed the effect of lncRNA ROR on proliferation, apoptosis and invasion of breast cancer cells

**Fig. 4:**
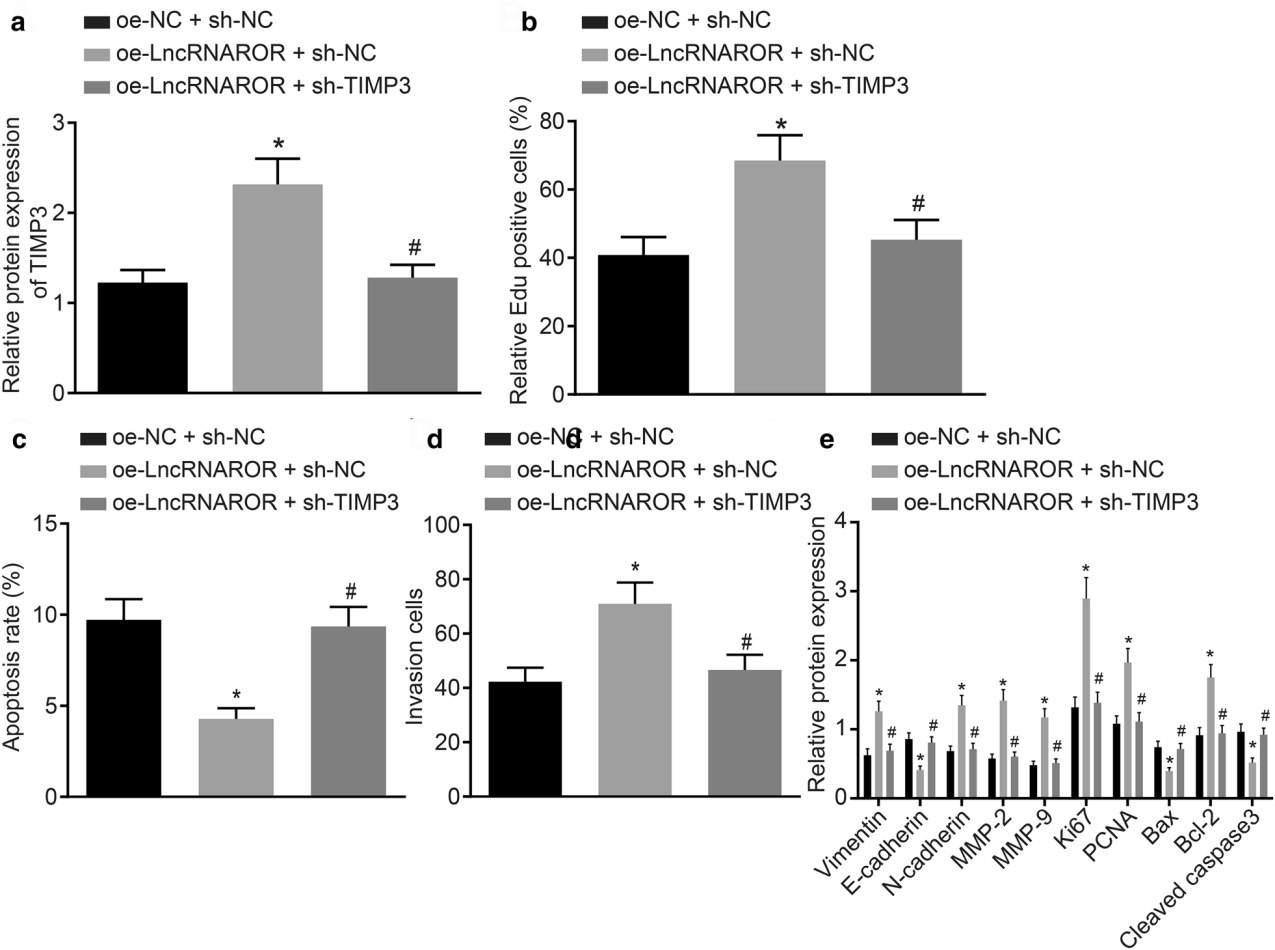
The effects of lncRNA ROR on the proliferation, invasion and apoptosis of breast cancer cells are reversed by TIMP3 silencing. **a** The expression of TIMP3 in MCF-7 cells co-transfected with oe-lncRNA ROR and/or sh-TIMP3 determined by Western blot assay. **b** The proliferation of MCF-7 cells co-transfected with oe-lncRNA ROR and/or sh-TIMP3 detected by EdU assay. **c** The apoptosis of MCF-7 cells co-transfected with oe-lncRNA ROR and/or sh-TIMP3 detected by flow cytometry assay. **d** The invasion of MCF-7 cells co-transfected with oe-lncRNA ROR and/or sh-TIMP3 detected by Transwell assay and the expression of TIMP3 of MCF-7 cells transfected with oe-lncRNA ROR and/or sh-TIMP3 determined by Western blot analysis. **e** The protein levels of Vimentin, N-cadherin, MMP-2, MMP-9, Ki67, PCNA, Bcl-2, E-cadherin, Bax and Cleaved caspase-3 in MCF-7 cells transfected with oe-lncRNA ROR and/or sh-TIMP3 determined by Western blot analysis. *p < 0.05 vs. that of the cells co-transfected with oe-NC and sh-NC. ^#^*p* < 0.05 vs. that of the cells co-transfected with oe-lncRNA ROR and sh-NC. The above data are measurement data and expressed as mean ± standard deviation. Comparisons among multiple groups are analyzed by independent sample *t* test. The experiment is repeated 3 times. MLL1, mixed-lineage leukemia 1; TIMP3, tissue inhibitors of metalloproteinase 3; NC, negative control; FISH, fluorescent in situ hybridization; DMSO, dimethyl sulfoxide; RIP, RNA immunoprecipitation; ChIP, Chromatin immunoprecipitation

Next, to ascertain the effect of lncRNA ROR on the biological function of breast cancer by regulating TIMP3, the MCF-7 cells were co-transfected with oe-NC and sh-NC, oe-lncRNA ROR and sh-NC or oe-lncRNA ROR and sh-TIMP3 with the results indicating that: compared with that of the cells co-transfected with oe-NC and sh-NC, the expression of TIMP3 was notably increased in the MCF-7 cells co-transfected with oe-lncRNA ROR and sh-NC, and meanwhile MCF-7 cell proliferation and invasion abilities were enhanced while apoptosis was notably reduced (Fig. 4a–e). The effects mediated by lncRNA ROR overexpression on the aforementioned were all reversed by sh-TIMP3 (Fig. 4a–e). The results suggested that  lncRNA ROR knockdown regulated proliferation, apoptosis and invasion of breast cancer cells by inhibiting TIMP3.

### LncRNA ROR depletion inhibits tumor growth of breast cancer

**Fig. 5:**
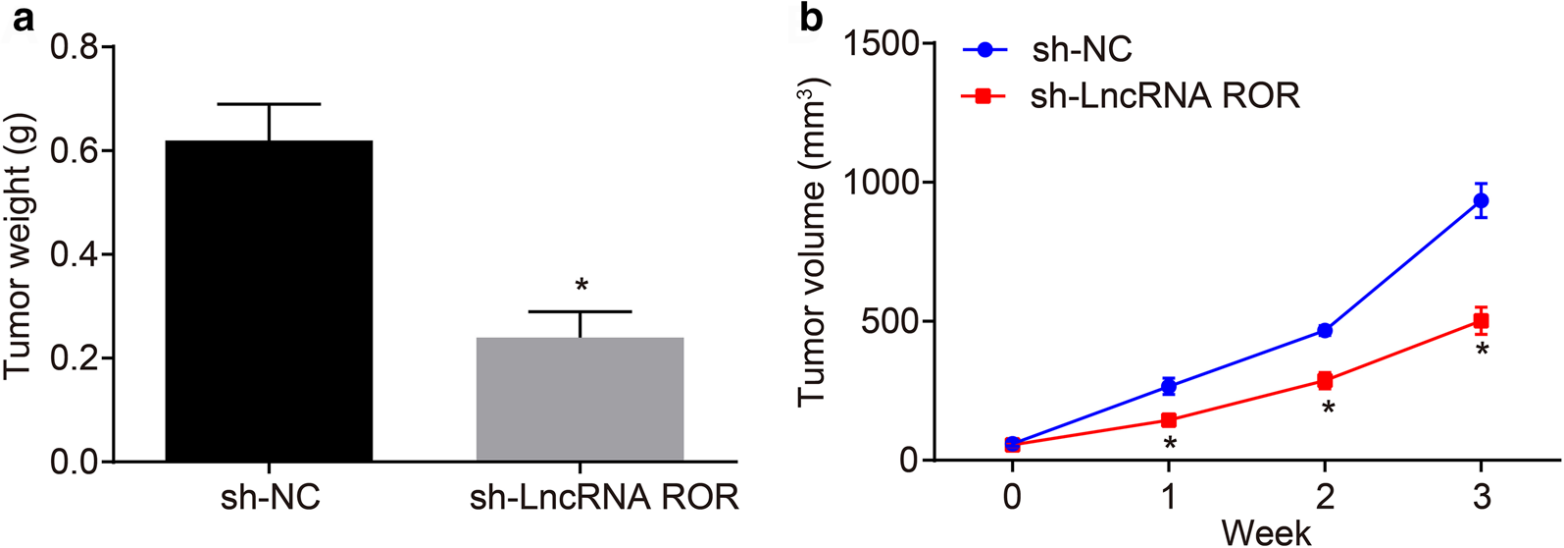
Silencing of lncRNA ROR induced inhibition of tumor growth in breast cancer. Nude mice were injected with cells treated with sh-lncRNA ROR. **a** Tumor weight of nude mice. **b** Tumor volume of nude mice. *p < 0.05 vs. mice treated sh-NC. n = 12. The above data are measurement data and expressed as mean ± standard deviation. Comparisons between two groups are analyzed by independent sample *t* test. Data at various time points are analyzed by repeated measures analysis of variance. Post hoc test is conducted using Tukey’s test. The experiment is repeated 3 times. TIMP3, tissue inhibitors of metalloproteinase 3; n, number

Finally, tumor xenograft in nude mice was performed to further characterize the role of lncRNA ROR in tumor growth in vivo. The results confirmed that the tumors had successfully formed in all the nude mice within a week, with the tumor volume progressively increasing with time. Silencing of lncRNA ROR triggered a reduced tumor volume and weight (Fig. 5a, b). The aforementioned findings demonstrated that lncRNA ROR silencing suppressed tumor growth in breast cancer.

## Discussion

**Fig. 6:**
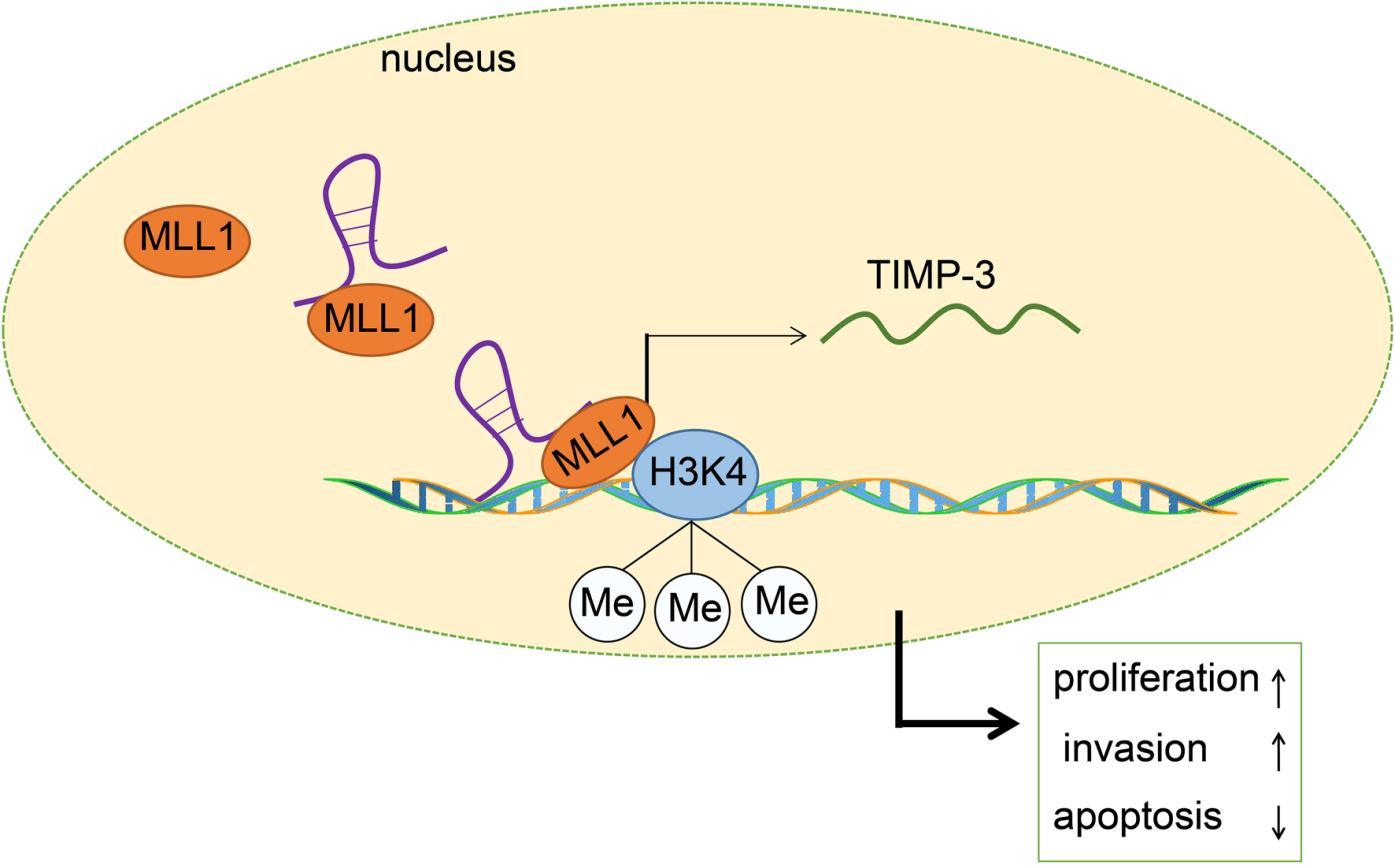
The mechanism depicts that lncRNA ROR is involved in breast cancer progression. LncRNA ROR recruits histone transmethylase MLL1 to TIMP3 promoter region to promote histone H3K4 methylation and up-regulate TIMP3 level, thus accelerating breast cancer progression

Breast cancer remains one of the most common malignancies, treatment for which remains a complex task [23]. At present, transcripts of several lncRNA have been implicated in the tumorigenesis of breast cancer [24]. Aberrant DNA methylation has provided fresh insight into the characterization of the molecular mechanisms associated with breast cancer [25]. Hence, the current study set out to elucidate the regulatory role of lncRNA ROR in breast cancer progression. Collectively, key observations made during the study suggest that lncRNA ROR silencing inhibited breast cancer progression by suppressing transmethylase MLL1 and TIMP3 (Fig. 6).

An initial finding in our study revealed that lncRNA ROR was upregulated in breast cancer. LncRNA ROR has been suggested to exhibit high levels of expression in non-small-cell lung cancer tissues with its high levels of expression highlighted in lymph node metastasis, positive distant metastasis and advanced TNM stage [26]. Consistent with our results, the expression of lncRNA ROR has been previously reported to be up-regulated in breast cancer tissues compared with adjacent tissues [27]. Moreover, the above studies indicate the high expression levels of lncRNA ROR in breast cancer. Besides, the non-triple negative breast cancer MCF-7 cells displayed the highest level of lncRNA ROR, while the more aggressive triple negative breast cancer MDA-MB-231 cells exhibited a relatively lower level of lncRNA ROR. In an attempt to ascertain the function of lncRNA ROR in breast cancer, breast cancer MCF-7 cells were transfected with sh-lncRNA ROR and oe-lncRNA ROR, respectively, the results of which indicated that silencing of lncRNA ROR triggered a decrease in the expression of Vimentin, N-cadherin, MMP-2, MMP-9, Ki67, PCNA and Bcl-2, while elevated levels of E-cadherin, Bax and Cleaved caspase-3, indicating inhibited cell proliferation and invasion and promoted apoptosis in breast cancer. The members of Bcl-2 family such as Bcl-2 and Bax represent a crucial communication network among proteins involved in the mediation of cellular apoptosis [28–30]. PCNA has been shown to stimulate diverse protein-DNA and protein–protein communications, while Ki67 has been emphasized in previous work as a cell proliferation marker and an attractive prognostic factor for breast cancer therapy [31, 32]. As zinc-dependent endopeptidases, the potential of MMPs from a therapeutic perspective has been highlighted in various diseases [33]. As a member of the intermediate filament protein family, Vimentin is highly expressed in multiple cancers including breast cancer [34]. N-cadherin and E-cadherin have been implicated in aggressive tumor behavior, tumor initiation, progression, metastasis and invasion [35, 36]. Cleaved caspase-3 has been identified as a promising therapeutic target for patients with several cancers such as gastric cancer [37]. Consistent with our findings, a previously reported research has suggested that lncRNA ROR silencing contributes to a decreased rate of proliferation and invasive ability in breast cancer MCF-7 cells [38]. Wang et al. concluded that lncRNA ROR silencing suppresses the invasion and proliferation of gastric cancer stem cells [39].

The findings of our study provide evidence illustrating that lncRNA ROR decoys transmethylase MLL1 to promote H3K4 methylation and up-regulate TIMP3, consequently promoting the progression of breast cancer. TIMPs serve as inhibitors of matrix metalloproteinases to prevent extracellular matrix from degradation [40]. Hartland et al*.* revealed that TIMP3 plays a distinct role in the progression of breast cancer, while suggesting that its deficiency inhibits early stage mammary tumorigenesis [22]. MLL1 is a member of SET1 family in H3K4 methyltransferases and the mutation of some family members is linked to cancer and developmental disorders [41]. Importantly, the mutation of MLL3 is of great significance for breast cancer development [42]. H3K4 methylation mediated by MLL1 plays a regulatory role in transcriptional initiation by RNA polymerase II [43]. Fan et al. asserted that lncRNA ROR serves as a promoter of tumorigenesis by inducing gene-specific histone methylation [12]. Gene promoter hypermethylation has been reported as a marker and mechanism of oncogenesis, while TIMP3 promoter methylation has been closely linked with tumor metastasis and invasion [44]. Interestingly, a relationship between TIMP3 hypermethylation, lymph nodes metastasis and high tumor grading in invasive breast ductal carcinoma has been speculated [45].

## Conclusion

Taken together, the key findings of our study provide evidence illustrating the role of lncRNA ROR as an oncogene in breast cancer via the recruitment of transmethylase MLL1 to the TIMP3 promoter region, elevating TIMP3 level (Fig. 6). These findings highlight the potential of lncRNA ROR as a promising marker for breast cancer prognosis and a therapeutic target for this disease. However, the research is still at the preclinical stage, and the role and mechanism of lncRNA ROR in breast cancer require further investigation. Thus, in future studies, a larger cohort and in vivo experiments should be conducted to further elucidate the underlying mechanism associated with lncRNA ROR, MLL1, H3K4 methylation and TIMP3 in breast cancer.

## Data Availability

The datasets generated/analyzed during the current study are available.

## Abbreviations

lncRNAs: Long non-coding RNAs
MLL1: Mixed-lineage leukemia 1
H3K4: Histone lysine 4
TIMPs: Tissue inhibitors of metalloproteinase
RT-qPCR: Reverse transcription quantitative polymerase chain reaction
TNM: Tumor node metastasis
DMEM: Dulbecco’s modified eagle medium
DAPI: 6-Diamidino-2-phenylindole
PBS: Phosphate buffered saline
